# Pediatric bronchogenic cyst complicated by atypical mycobacterium infection: a case report

**DOI:** 10.4076/1757-1626-2-8070

**Published:** 2009-09-10

**Authors:** Stacy A Frye, James M DeCou

**Affiliations:** 1 Grand Rapids Medical Education Center Pediatric Residency Program, Michigan State University, Helen DeVos Children’s Hospital330 Barclay Avenue NE, Suite 300, Grand Rapids, MI 49503USA; 2 Department of Pediatric Surgery, Helen DeVos Children’s Hospital330 Barclay Avenue NE, Suite 202, Grand Rapids, MI 49503USA

## Abstract

**Introduction:**

Bronchogenic cysts are lesions of congenital origin derived from the primitive foregut and are the most common primary cysts of the mediastinum. They are most frequently unilocular and contain clear fluid. Respiratory distress is the most common presentation in pediatric patients, manifested by recurring episodes of cough, stridor, wheezing and retractions.

**Case presentation:**

We report the first pediatric case of bronchogenic cyst complicated by atypical *Mycobacterium* infection. This case describes a 13-year-old Caucasian American female with a large cystic lesion and extensive pulmonary involvement. Pathology studies revealed necrotizing granulomatous inflammation, multiple nodules, and acid-fast bacilli. She was successfully treated with surgical excision and a six-week course of clarithromycin, rifampin and ethambutol. Other unusual aspects of this case include multilocular intraparenchymal cyst appearance, its turbid drainage, and late symptom onset.

**Conclusion:**

Bronchogenic cyst should be included in the differential diagnosis of a child with cough, dyspnea, and fever. Although rare, we stress the importance of keeping mycobacterial infection in mind in cases of an infected cyst. Acid-fast culture should be done on sputum and cyst contents. Due to the frequency of negative cultures, stains should also be performed on resected cyst specimens. Antibiotic therapy should be considered and administered based on the extent of infection. All symptomatic or enlarging cysts warrant surgical excision. Prophylactic removal of asymptomatic cysts is recommended due to higher rates of perioperative complications once cysts become symptomatic.

## Introduction

Bronchogenic cysts are lesions of congenital origin derived from the primitive foregut. They form due to ectopic budding of the foregut during the first trimester. Epithelial cells of the developing trachea and lung are pinched off and grow separately from the airways. Bronchogenic cysts are most commonly mediastinal, unilocular and contain clear fluid. Foregut cysts, including bronchogenic cysts, account for 10-18% of all mediastinal masses in pediatric patients. Clinically, most bronchogenic cysts are symptomatic and occur in infancy or early childhood. Respiratory distress is the most common presentation, manifested by recurring episodes of cough, stridor, and wheezing. Other diagnoses to consider with regard to cystic mediastinal lesions in the pediatric population include neurogenic tumors, foregut cysts, congenital lobar emphysema, cystadenomatous malformation, pulmonary sequestration and simple lymphatic cysts.

## Case presentation

A 13-year-old Caucasian American female presented with a two month history of right-sided back pain and five days of intermittent fever. The pain was worse on inspiration and made sleeping difficult. She denied wheezing, chest pain, or cough. She continued daily participation in competitive sports. Previous trials of antibiotics and an inhaled bronchodilator for presumed exercise-induced asthma were unsuccessful.

**Figure 1.**
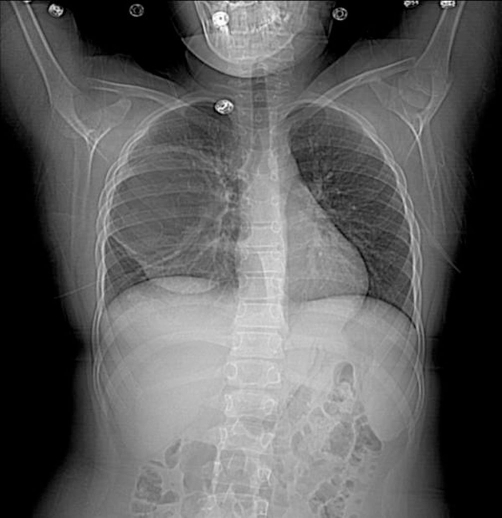
Initial chest X-ray revealing bronchogenic cyst in the posterior right middle lobe (10 × 10 × 8 cm^3^).

**Figure 2.**
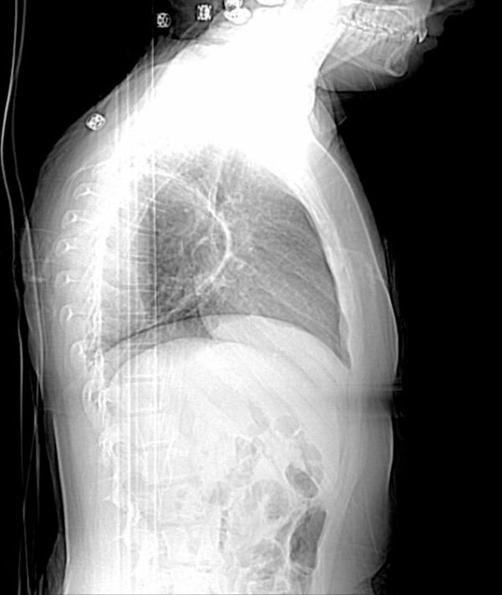
Initial lateral view chest X-ray.

**Figure 3.**
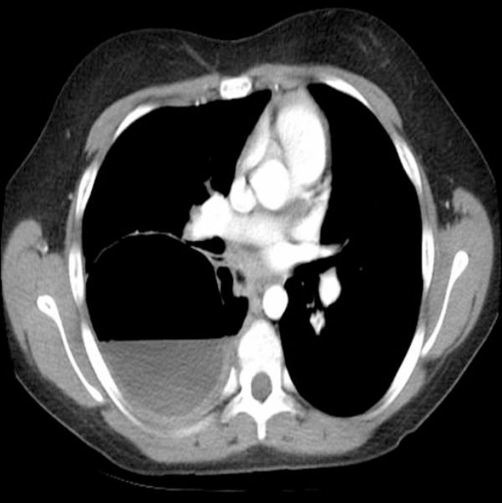
Chest CT shows bronchogenic cyst extending the entire width of the right hemithorax and approximately 50% full of fluid.

Chest X-ray (CXR) showed a large cyst (10 × 10 × 8 cm^3^) in the posterior right lung (Figures 1 and 2). Computerized tomography (CT) scan showed a large cystic lesion arising entirely within the right lower lobe and extending the width of the hemithorax (Figure 3). There was an air-fluid level occupying ~50% of the cavity. She was diagnosed with a multilocular bronchogenic cyst. She was briefly hospitalized and discharged on azithromycin with plans to resect the cyst in one month.

**Figure 4.**
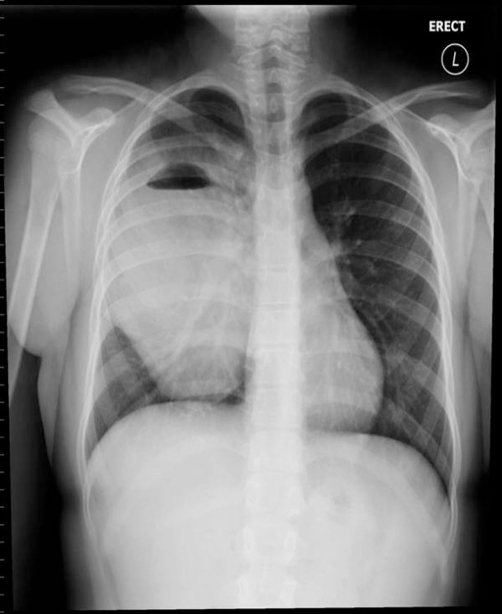
Substantial bronchogenic cyst, 16 × 9 × 11 cm^3^, over 95% full of fluid.

**Figure 5.**
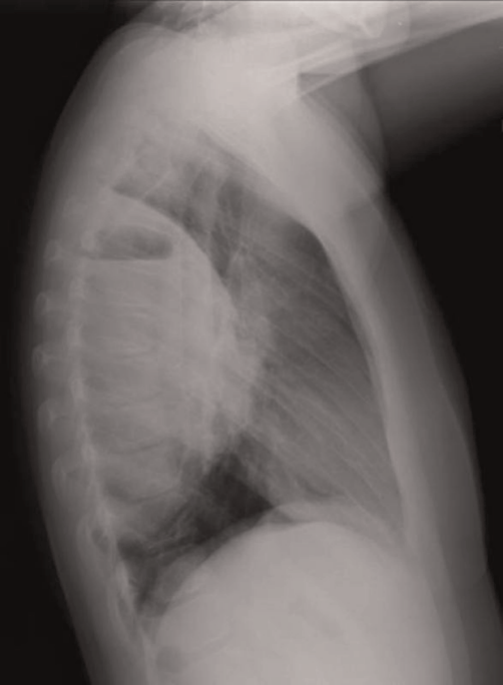
Lateral chest X-ray revealed opacification along superior margin of cyst.

Severe cough, fever, and chills prompted readmission after 3 weeks of antibiotic therapy. CXR and CT showed cyst enlargement (16 × 9 × 11 cm^3^) with over 95% fluid (Figures 4 and 5). She was started on ampicillin/sulbactam. Percutaneous drain placement yielded a large volume of turbid fluid. Aerobe, anaerobe and fungal studies of the fluid were negative. Resection was postponed due to significant inflammation surrounding the cyst cavity. She was discharged on a seven day course of amoxicillin/clavulanate.

Following six weeks of cyst drainage, a thoracoscopic right lower lobectomy was performed. Extensive inflammation and induration made dissection of the lower lobe and pulmonary vessels challenging. Fibrinoid adhesions extended to the pleural surface. Operative time was 418 minutes.

**Figure 6.**
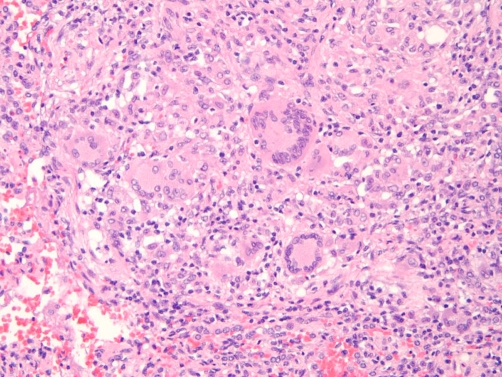
Pathology specimen with multinucleated giant cells.

**Figure 7.**
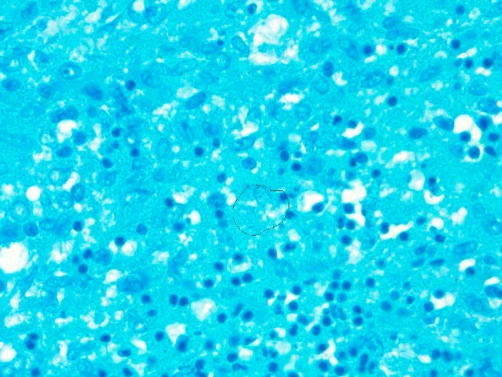
Acid fast stain showing occasional acid fast bacilli.

**Figure 8.**
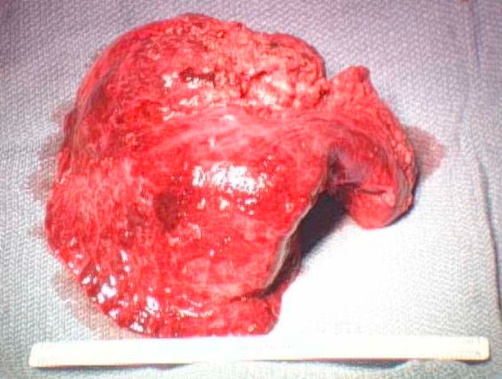
Gross specimen of right lower lobe: Approximately half of the lobe was indurated and 95% of surfaces showed nodular involvement. Sectioning through indurated region revealed diffuse nodules up to 3.3 cm. Nonindurated lung showed small nodules with miliary appearance.

Surgical pathology showed diffuse necrotizing granulomatous inflammation with acid-fast bacilli and multiple nodules up to 3.3 cm in diameter (Figures 6 and 7). 95% of the pleural surface had nodular involvement (Figure 8). Areas of non-indurated lung also showed small nodules with a miliary appearance. Inflammation was present at the bronchovascular margins, hilar nodes, and distal lung.

The patient had no history of tuberculosis exposure, foreign travel or immunodeficiency. There was no family history of tuberculosis or respiratory disease. Based on the acid-fast bacilli identified on pathology stain, fluid drained from her chest tube was sent for acid-fast bacilli culture and smear. *Mycobacterium* was not isolated. It was determined that the source of the atypical mycobacterial infection was likely colonizing mycobacteria from her oropharynx that became entrapped in the cyst. A six-week course of clarithromycin, rifampin, and ethambutol was prescribed to treat any remaining organisms.

At two-month follow-up, she had minimal pulmonary symptoms and inflammatory markers were improved. Erythrocyte sedimentation rate (normal: 0-15) and C-reactive protein level (normal: 0-10) decreased from 88 and 173 during her hospitalization, to 10 and 3.6, respectively. At four-month follow-up, she had resumed competitive sports and had no evidence of ongoing infection.

## Discussion

This case highlights a unique presentation of infected bronchogenic cyst after substantial cyst growth. Unusual aspects include the late onset of symptoms, multilocular intraparenchymal cyst appearance, turbid drainage, extensive nodularity, necrotizing granulomatous inflammation, and atypical *Mycobacterium* infection. Although comorbid infection is not uncommon, causative organisms are typically *Haemophilus influenzae* [1,2], and *Streptococcus pneumoniae* [3]. Cases of *Streptococcus pyogenes* [4], *Escherichia coli* [5], and *Salmonella enteritidis* [6], have been reported. However, only four cases of bronchogenic cyst with *Mycobacterium* infection have been documented [7-9].

Three of the *Mycobacterium*-infected cases are adult patients. Lin et. al reported a 39-year-old female with bronchogenic cyst complicated by *Mycobacterium avium* infection [7]. The organism was identified by genetic sequencing of biopsied lung tissue. Sputum acid-fast stain and mycobacterial cultures were negative. Liman et al. reported two adult cases: a 20 year-old male with *Mycobacterium* identified in a right lower lobe specimen but with negative sputum culture, and a 32-year-old female with *Mycobacterium* isolated in a sputum culture but a negative microscopic exam and cyst fluid culture [8].

The only documented pediatric case, a 9 year-old female with a 6 cm right lower lobe bronchogenic cyst, was reported by Houser et al [9]. She underwent lobectomy; Kinyoun stain of the cyst specimen showed *Mycobacterium.* Sputum culture and acid-fast bacilli stain were negative. Tuberculin skin test was positive. Comorbid infection with *Mycobacterium tuberculosis* was suggested, but they were unable to isolate an organism. Treatment consisted of four months of rifampin and two years of isoniazid with pyridoxine.

This is the first documented pediatric case of bronchogenic cyst infected with atypical *Mycobacterium.* Her presentation is noteworthy, given the substantially greater size of the cyst (16 × 9 × 11 cm), extensive pathologic findings, and success with a different antibiotic regimen.

Bronchogenic cyst should be included in the differential diagnosis of a child with cough, dyspnea, and fever. Although rare, we stress the importance of keeping mycobacterial infection in mind in cases of an infected cyst. Acid-fast culture should be done on sputum and cyst contents. Due to the frequency of negative cultures, stains should also be performed on resected cyst specimens. Antibiotic therapy should be considered and administered based on the extent of infection. All symptomatic or enlarging cysts warrant surgical excision. Prophylactic removal of asymptomatic cysts is recommended due to higher rates of perioperative complications once cysts become symptomatic [10]. We raise the question of whether earlier CXR is indicated to rule out bronchogenic cyst, particularly when patients do not improve after trials of watchful waiting, antibiotics, and bronchodilators for other possible respiratory diagnoses.
